# The Sexual Development and Education of Preschool Children: Knowledge and Opinions from Doctors and Nurses

**DOI:** 10.1007/s11195-015-9393-9

**Published:** 2015-01-22

**Authors:** Meltem Kurtuncu, Latife Utas Akhan, İbrahim Murat Tanir, Hicran Yildiz

**Affiliations:** 1 Department of Pediatric Nursing, Zonguldak School of Nursing, Bülent Ecevit University, Zonguldak, Turkey; 2 Sultanbeyli State Hospital, Istanbul, Turkey; 3 Division of Nursing, Health Science Faculty, Uludag University, Bursa, Turkey

**Keywords:** Children, Sexual development, Sexual education, Preschool period, Turkey

## Abstract

This descriptive study was carried out in order to determine the knowledge levels and attitudes of doctors and nurses regarding children’s sexual development and sex education. The study was conducted with doctors and nurses who work at various clinics of two state hospitals located in the province of Istanbul. The data collection tool consisted of 58 questions. The Statistical Program for the Social Sciences, version 18.0 (SPSS 18.0) was used for data analysis. It was determined that females comprised the majority of the respondents (61 %) and were over 36 years of age (54.1 %) (37.81 ± 8.82). Of the participants in the study, 63.5 % had bachelor’s degrees and 62.1 % were medical doctors. It was determined that the number of correct responses given by the respondents regarding some behaviors observed in children aged between 3 and 6 years and children’s sexual development and sex education showed significant differences according to age group (*p* = 0.007), marital status (*p* = 0.004), the status of having children (*p* = 0.004), educational status (*p* = 0.005) and occupation (*p* = 0.000). However, in a review of the study findings, it was observed that culture had an important impact on sex-related approaches and that embarrassment and shyness is very common.

## Introduction

The preschool period is the time when a child meets, learns about, and communicates first with his close environment and family and then with the extended family and the whole environment [1, 2]. The period particularly between the ages of 0–6, which is known as the early childhood period (preschool period), is a very critical period where learning is fastest, the child is affected the most from environmental factors, and the chance is highest that the child will maintain acquired attitudes, behavior and habits in the following years [1]. In this period when the basis of the child’s personality is formed, the child needs guidance from a source knowledgeable about areas of development. One of those areas is the sexual development of the child. As with many other subjects, placing importance on sexual education in this period has many benefits in terms of development [3–5].

The concept of sexuality is generally attributed to adolescence and kept separate from childhood. However, sexuality does not emerge suddenly during adolescence; it starts from the moment a baby is born [6]. In adolescence, knowledge and attitudes about sexuality change under the impact of environmental factors, going beyond the individual’s own thoughts and perceptions [7, 8]. Parents, teachers, families, neighbors and the media all have important roles in the sexual education of children and give children sexual education from birth without even noticing that they are doing so [4, 8–13]. Studies have confirmed that sexual education is a lifelong process that starts at birth [14, 15].

Bulut [16] stated that the sexual education of children has been long neglected, most probably because children do not have the ability to reproduce. Artan [17] has suggested that the term “sexual education” primarily conjures up the idea of reproduction and thus, having sex. However, when the definition of sexual identity is considered, it is very clear that the context of sexual education should be very different [3].

There are different ideas on whether or not preschool children should receive sexual education, which topics such an education would include, and at what age such education should be started. In particular, parental awareness of the role they play in the personal development of their children will have a positive effect on their children’s sexual development. A child who receives sexual education in phases in a manner appropriate to his/her age would be expected to be more stable in his/her relationships with the opposite sex in later life [15].

There have not been sufficient studies in Turkey on the sexual education of children. Efforts aiming to raise awareness and provide knowledge to families have also been insufficient. It can be seen that there are many more studies on the subject in other countries where these topics are included in educational programs [18, 19].

Doctors and nurses who work in healthcare are in an important position to achieve the early diagnosis of high-risk children and families. Health professionals working in hospital units are the first to encounter children and families [20]. An important role falls on the shoulders of health professionals in educating children about sexual development and in preparing and handing out booklets to families via family health centers [21]. Thus, it is important to know the extent of knowledge of doctors and nurses and their attitudes on the sexual development and education of children.

This study was performed in order to determine the knowledge, attitudes, and opinions of doctors and nurses working in health care who are in a unique position to reach parents regarding the sexual development and education of their children.

## Materials and Methods

### Study Group and Sample Size

This descriptive study was carried out in order to determine knowledge levels and attitudes of doctors and nurses regarding children’s sexual development and sex education. The study was conducted with doctors and nurses working at the various clinics of two state hospitals located in the province of Istanbul. A total of 138 doctors and 150 nurses work at these hospitals. The study was completed with the participation of 104 doctors and 55 nurses since some of them were on vacation, were absent due to sickness, or did not wish to participate in the study.

### Collection of Data

The data collection tool consisted of 58 questions and was prepared by the researcher based on published literature and the opinions of two experts [2, 3, 5]. The questionnaire consisted of three sections: The first section included seven questions about the demographic characteristics of the doctors and nurses; the second section included five questions about the knowledge of the doctors and nurses regarding children’s sexual development and sex education. The third section included 46 items on some sexual behaviors of children, children’s sexual development, and sex education. The choices in the third section were “True”, “False”, and “I do not know”. A test run of the questionnaire was carried out with ten people. Data pertaining to the participants taking part in the test run were excluded from the study.

### Implementation of the Data Collection Tool

This study was conducted between October and November 2013. The researchers visited clinics during the night and day shifts and gave the questionnaire to doctors and nurses who agreed to participate in the study. The aim of the study was explained to the participants, who completed the forms individually. Filling out the questionnaire took an average of 15 min. The completed forms were received in sealed envelopes.

### Assessment of Data

The Statistical Program for the Social Sciences Version 18.0 (SPSS 18.0) was used for data analysis. In addition, we calculated percentages and averages and performed the Kruskall Wallis-H, Mann–Whitney U and Tukey HSD tests for statistical analysis.

### Ethical Aspects of the Study

Written permission was obtained from the hospitals where the study was conducted. In the light of the principle of autonomy, “informed consent” was obtained from the doctors and nurses after explaining the study’s aim, plan, and its expectations from the participants. Participation in the study was voluntary. We told the participants that they were free to participate or not participate, that they could withdraw from the study at any point and that they had the right to abstain from providing information. The “loyalty-privacy” principle was upheld by reassuring the participants that their personal information would not be revealed to anyone other than the researchers or that no one else would be permitted others to access this information.

### Findings

In this study, which was conducted in order to determine knowledge levels, attitudes, and opinions regarding sex education for children among doctors and nurses working in health services who are in an important position to reach parents with regard to children’s sexual development and sex education, we reached the following results.

### Sociodemographic Characteristics

It was determined that the females comprised the majority of the respondents (61 %) and were over the age of 36 years (54.1 %) (37.81 ± 8.82). Of the respondents, 81.1 % were married and 89.4 % were members of a nuclear family. Among the married participants, 80.5 % had children and most (41.4 %) had two children. Another 63.5 % had bachelor’s degrees and 62.1 % were medical doctors (Table 1).

**Table 1:** Distribution of socio-demographic characteristics among cases (N = 159)

Socio-demographic characteristics	n	%
Gender		
Female	97	61.0
Male	62	39.0
Age		
25 and ↓	7	4.4
26–30 years	26	16.4
31–35 years	40	25.2
36 and ↑	86	54.1
Age		
($\bar{X}$ ± SD years)	37.81 ± 8.82	(Range: 18–62)
Marital status		
Married	129	81.1
Single	26	16.4
Divorced	4	2.5
Family type		
Nuclear family	135	84.9
Extended family	20	12.6
Single parent	4	2.5
Do you have children?		
Yes	128	80.5
No	31	19.5
Number of children (n = 128)		
1	49	38.3
2	53	41.4
3	22	17.2
4 and more	4	3.1
Educational status		
Vocational school of health	12	7.5
Associate of science	10	6.3
Bachelor’s degree	101	63.5
Other	36	22.6
Occupation		
Doctor	104	65.4
Nurse	55	34.6

### Sexual Education

Of the respondents, 93.1 % thought that it is necessary to provide sex education for children. The majority (54.7 %) stated that sex education for children should start at the ages of 7–12 years and 30.8 % thought that parents should provide this education. Another 6.3 % had attended a training program on sex education for children and 30 % reported that they received this training during undergraduate study.

### Attitudes Toward Sexual Education

Of the respondents, 85.2 % reported that they had waited for their children to ask questions in order to inform them about sexuality and 70.3 % stated that they talked to their children on any topic related to sexuality. Among the study participants, the most common topic talked about with children was “bodily differences between genders” and the topic least talked about was “masturbation” (Table 2).

**Table 2:** Distribution of opinions and approaches regarding sexuality among cases (N = 159)

Thoughts about sex education	n	%
Do you think it is necessary to provide sex education for children?		
Yes	148	93.1
No	11	6.9
At what age do you think sex education should be started?		
0–3 years	2	1.3
4–6 years	63	39.6
7–12 years	87	54.7
13–18 years	7	4.4
Who should provide sex education for your child?		
Mother–father	49	30.8
School	2	1.3
An expert	65	40.9
Parents and schools	29	18.2
Parents, school and experts	13	8.2
Parents and experts	1	0.6
Did you receive training on children’s sex education before?		
Yes	10	6.3
No	149	93.7
From whom did you receive this training and how long was the training program? (n = 10)		
From an expert friend for a short period	2	20.0
During undergraduate study	3	30.0
During my specialization in medicine	1	10.0
From a private institution for 6 months	1	10.0
From a private center for a month	1	10.0
From a psychiatrist friend for a short period	1	10.0
From a psychologist	1	10.0
Do you wait for your child to ask questions in order to provide information on sexuality?		
I wait for him/her to ask questions	109	85.2
I do not wait for him/her to ask questions	19	14.8
Have you talked to your child about any sexuality related topic?		
Yes	90	70.3
No	38	29.7
Which topics did you talk with your child? (n = 90)^a^		
Bodily differences between genders	90	100
Pregnancy and birth	63	70
Reproduction	71	78.9
Slang and swear words	37	41.1
Masturbation	10	11.1
Sexual abuse	16	17.8
Health and hygiene rules	79	87.8
Sexual curiosity and games (marital games and playing doctor)	54	60.0
AIDS	13	14.4
Other sexually transmitted diseases	15	16.7

^a^More than one choice was marked

When we examined the distribution of the opinions of the respondents regarding some behaviors observed in children between the ages of 3–6 years and the topic of children’s sexual development and sex education (Table 3), we found that the respondents found the statement “Children aged between 5 and 6 years may try to touch their mothers’ or other women’s breasts” the most accurate statement (96.2 %) and that the statement “Sexuality and sex education starts during adolescence” was perceived as the most inaccurate statement (71.1 %). The mean total accurate response score was found to be 33.15 ± 5.70.

**Table 3:** Distribution of opinions regarding some behaviors observed in children aged between 3 and 6 years, sexual development and sex education (N = 159)

	True		False		I do not know	
	n	%	n	%	n	%
Children who are 6 years old may try to perform sexual behaviors they see on (kissing, sleeping together, etc.)	150	94.3	9	5.7	–	–
Sexual development and sex education start during the maternal period and continues through the life span	150	94.3	9	5.7	–	–
Children aged between 5 and 6 years may try to touch their mothers’ or other women’s breasts	153	96.2	6	3.8	–	–
The first information on sexuality should be provided by parents for their children	150	94.3	9	5.7	–	–
Children’s and adults’ sexual behaviors are not similar and are not performed for the same purposes	143	89.9	13	8.2	3	1.9
Children aged between 5 and 6 years sometimes may try to kiss other children or peers by force	139	87.4	17	10.7	3	1.9
Children should be oriented toward choosing gender appropriate clothes. Toys, colors, etc.	139	87.4	16	10.1	4	2.5
Children aged between 5 and 6 years may try to undress other children or peers	122	76.7	23	14.5	14	8.8
Sex education should be provided at kinder garden	127	79.9	19	11.9	13	8.2
Girls’ effort to look like their mothers and boys’ effort to look like their fathers is called identification and this process takes place in order to adopt sexual identity during 3–6 years of age	130	81.8	17	10.7	12	7.5
Children aged between 3 and 6 years may try to Show their genitals to other children or peers or adults	121	76.1	28	17.6	10	6.3
Sexual abuse is an adults’ sex-oriented approach toward a child and involves sex related acts. expressions and thoughts	127	79.9	19	11.9	13	8.2
It is important for the development of children aged between 5 and 6 years to know names of body parts and their functions	131	82.4	19	11.9	9	5.7
Children do not have sexual instincts	37	23.3	97	61.0	25	15.7
Children aged between 5 and 6 years can define/explain their gender according to cultural characteristics	137	86.2	13	8.2	9	5.7
Children do not need sex education before the age of 5–6	123	77.4	29	18.2	7	4.4
It is normal for children aged between 5 and 6 years to play doctor and marital games and during these games children can take off their clothes and inspect each other’s body	144	90.6	11	6.9	11	6.9
Children start to ask questions about the father’s role and contribution regarding birth after the age of 4	129	81.1	15	9.4	15	9.4
Children aged between 5 and 6 years can make gender appropriate toy choices	149	93.7	7	4.4	3	1.9
Children aged between 5 and 6 years may sometimes use sex related slang words	137	86.2	15	9.4	7	4.4
Sexuality is a healthy and natural part of life that starts at birth and continues through the life span	142	89.3	14	8.8	3	1.9
Children ask questions about sexuality (questions about birth and pregnancy such as “where do babies come from?”) before the age of 6 (at ages 2.5–3 in particular)	135	84.9	21	13.2	3	1.9
Sexuality and sex education start during adolescence	41	25.8	113	71.1	5	3.1
Sucking the mother’s breast is the first sexual experience of the baby	67	42.1	76	47.8	16	10.1
Parents, teachers and caregivers should be in cooperation in terms of sex education	133	83.6	16	10.1	10	6.3
Children aged between 5 and 6 years are curious about other people’s rest room and bathing activities and may try to observe them.	137	86.2	13	8.2	9	5.7
Babies touch their genitals when their diaper is changed and they enjoy this. This behavior is the beginning of taking pleasure	126	79.2	21	13.2	12	7.5
Children aged between 3 and 6 years take pleasure in touching their own genitals and being naked	127	79.9	19	11.9	13	8.2
Children should be punished when they use slang words with sexual content	81	50.9	60	37.7	18	11.3
Sex education should be provided when children start to ask questions about sexuality	129	81.1	16	10.1	14	8.8
Children ask questions about the differences of girls’ boys’ bodies before the age of 6 (at ages 2.5–3 in particular)	129	81.1	16	10.1	14	8.8
Children who are 6 years old choose clothes appropriate for their gender	130	81.8	15	9.4	14	8.8
Children should learn to name their body parts in an appropriate and decent fashion instead calling these body parts with their real names	130	81.8	21	13.2	8	5.0
Children aged between 5 and 6 years choose gender appropriate roles in games	137	86.2	11	6.9	11	6.9
Parents are more responsible in providing sex education for children compared to schools	134	84.3	13	8.2	12	7.5
In children aged between 5 and 6 years, mothers should answer girls’ questions on sexuality and fathers should answer boys’ questions	125	78.6	25	15.7	9	5.7
Masturbation is a part of childhood development	64	40.3	73	45.9	22	13.8
The effect of gender is observed in friend choices during 3 years of age	138	86.8	11	6.9	10	6.3
Some sexuality related questions of children aged between 5 and 6 years can be ignored	132	83.0	23	14.5	4	2.5
Family’s social system provides a model for children in their relationships with other people and teaches them many things	150	94.3	6	3.8	3	1.9
It is not appropriate for girls and boys who are 6 years old to sleep in the same room in terms of their development	134	84.3	20	12.6	5	3.1
Only girls can be subject to sexual abuse	46	28.9	109	68.6	4	2.5
Children aged 5–6 years can bathe with their peers and parents	116	73.0	37	23.3	6	3.8
Most children aged less than 6 years and at age 3 in particular are curious and talkative about their own and other people’s bodies, experiences and feelings	146	91.8	11	6.9	2	1.3
Children’s love for their caregivers, teachers or parents can sometimes be perceived as a romantic feeling	147	92.5	8	5.0	4	2.5
If you encounter a scene that includes sexuality while you are watching television with your child possible solutions for this situation are turning off the television, switching the channel or closing your child’s eyes	137	86.2	12	7.5	10	6.3
Total number of correct answers	$\bar{X}$ ± SD		33.15 ± 5.70		Range = 17–40	

It was determined that the number of correct responses given by the respondents regarding some behaviors observed in children between the ages of 3–6 years and children’s sexual development and sex education showed significant differences according to age group, marital status, the status of having children, educational status, and occupation (Table 4).

**Table 4:** Distribution of differences in the number of correct responses by sociodemographic characteristics (N = 159)

Socio-demographic characteristics	$\bar{X}$ ± SD	Level of significance
Gender		U = 2,787.50
Female	33.57 ± 5.66	Z = −0.782
Male	32.50 ± 5.74	*p* = 0.434
Age group		KW = 12.000
25 vs. ↓	27.00 ± 3.55	*p* = 0.007
26–30 years	32.34 ± 5.79	
31–35 years	34.85 ± 5.88	
36 vs. ↑	33.11 ± 5.40	
Marital status		KW = 11.02
Married	33.58 ± 5.70	*p* = 0.004
Single	30.30 ± 5.05	
Divorced	37.75 ± 2.62	
Family type		
Nuclear family	32.80 ± 5.81	
Extended family	34.60 ± 4.87	KW = 5.159
Single parent	37.75 ± 2.62	*p* = 0.076
Do you have children?		U = 1,330.50
Yes	33.85 ± 5.51	Z = −2.866
No	30.25 ± 5.62	*p* = 0.004
Number of children (n = 128)		
1	33.57 ± 6.05	
2	33.86 ± 5.54	KW = 4.641
3	35.40 ± 3.93	*p* = 0.200
4 and more	28.75 ± 2.75	
Educational status		
Vocational school of health	27.41 ± 4.94	
Associate of science	30.60 ± 5.58	U = 2,094.50
Bachelor’s degree	35.83 ± 4.30	Z = −2.796
Other	28.27 ± 4.46	*p* = 0.005
Occupation		
Doctor	33.89 ± 5.69	KW = 61.54
Nurse	31.76 ± 5.50	*p* = 0.000

The number of correct answers is less in the single respondents than in the married and divorced. The number of correct answers in the age group 25 and under is less than in the age groups 31–35 and 36 and above. The number of correct answers is higher in the group of university graduate respondents compared to other graduates.

The number of correct responses given by the respondents regarding some behaviors observed in children of the ages 3–6 and children’s sexual development and sex education showed significant differences according to whether or not the respondents thought it was necessary to provide children with sex education and whether or not they talked to children about any topic related to sexuality (Table 5).

**Table 5:** Distribution of differences in the number of correct responses by opinions and approaches regarding sex education (N = 159)

Thoughts about sex education	$\bar{X}$ ± SD	Level of significance
Do you think it is necessary to provide sex education for children?		U = 527.50 Z = −1.96 *p* = 0.050
Yes	33.43 ± 5.65	
No	29.45 ± 5.33	
Did you receive training on children’s sex education before?		U = 499.50 Z = −1.75 *p* = 0.079
Yes	30.80 ± 5.30	
No	33.31 ± 5.71	
Do you wait for your child to ask questions in order to provide information on sexuality?		U = 1,421.00 Z = −0.433 *p* = 0.665
I wait for him/her to ask questions	33.10 ± 5.66	
I do not wait for him/her to ask questions	33.45 ± 6.08	
Have you talked to your child about any sexuality related topic?		U = 814.50 Z = −8.00 *p* = 0.000
Yes	36.59 ± 3.14	
No	28.55 ± 5.09	

## Discussion

In a review of studies conducted between 1975 to the present on the topic of preschool children in Turkey, we determined that topics related to sexual development and sex education were never mentioned in earlier studies. It was seen, however, that a small number of studies gradually emerged after the 1980s and the number of these studies continued to increase [22].

### Sexual Education

In the present study, it was found that 93.1 % of the respondents thought that it was necessary to provide children with sex education and the majority (54.7 %) stated that sex education for children should start at the ages of between 7 and 12 years. Tuğrul and Artan [23] reported that 7.5 % of mothers believed that the sex education of children should start at ages 0–3 while 17.4 % believed this should happen at 3–6 years of age, 39.6 % pointed to the elementary school years as the appropriate time, and 27.8 % thought that the high school years are the best time. In a study El Shaieb and Wurtele [24] conducted with parents, it was determined that families did not talk to their children about sexuality before the ages of 5–7 years. Ages 7–12 correspond to the period that children start to attend elementary school. However, in the literature, it has been reported that providing sex education at early ages would contribute to the formation of healthier sex lives during adulthood [25]. When we look at Freud’s developmental stages, we see that sexuality is present at birth and that during the phallic phase (3–6 years of age) behaviors such as masturbation are among the dominant functions of the phase [26]. Experiences pertaining to the earlier stages of life are very influential in the development of a sexual identity. Sexual experience and sexual perception are affected by personal experience and contribute to the development of gender identity [8, 9, 27].

When we examined the responses given to the question “Who should provide sex education for your child?” that was related to the respondent’s opinions and approaches regarding sex education, we observed that 40.9 % answered as “An expert should provide sex education for my child” and 30.8 % of the cases responded as “Parents should be the ones to provide their children with sex education.” In their study with mothers, Tuğrul and Artan [23] found that 89.8 % of mothers believed that mothers should be the ones to give a child sex education. In a qualitative study conducted with parents by Walker [28], some parents reported that they wanted experts of sexual health education and health professionals to provide their children with sex education. This result is in parallel with our study. Our study revealed that, because the respondents were doctors and nurses, they recognized the importance of having a specialist be involved in every kind of education in the area of healthcare. It was observed furthermore that some of the respondents who had themselves received education in this area were the ones to suggest that “experts should provide sex education.” On the other hand, the second most common response that was received was that “parents should provide sex education.” This response is consistent with the results of Tuğrul and Artan’s study. The small number of subjects who received this kind of education indicates that cultural mores still have an influence on people in this domain.

### Attitudes Towards Sexual Education

In the study, it was determined that the most talked about topic with children was “bodily differences between genders” (100 %), whereas the least talked about topic was “masturbation” (41.1 %) among the respondents. In a study by El Shaieb and Wurtele [24] conducted with families, it was found that parents had the most difficulty in talking about masturbation and nocturnal ejaculation with their children. In a study by Diiorio et al. [29] conducted with parents, it was determined that the topic families talked least about with their children was masturbation (8–40 %). These results support our study findings.

In the study, 85.2 % of the respondents reported that they waited for their children to ask relevant questions before providing them with information on sexuality. In a qualitative study by Walker [28] conducted with families, some mothers reported that they answered questions about sexuality only if children asked such questions. Family counselors state that parents should initiate conversations about human anatomy during the preschool period, and about reproduction and birth in particular by age 2 years without waiting for their children to ask questions about sexuality [18]. In our society, bringing up any topic related to sexuality is considered shameful, sinful and subjects that are avoided, not spoken out loud or mentioned in any way [3, 26]. Studies have shown that waiting for a child to ask questions before starting with sex education leads to feelings of shyness and embarrassment because of the cultural pressures that exist. Parents need to be educated before this situation can change. The fact that there were so few respondents in our study who had received education in this area even though they were doctors and nurses is not surprising.

It was found in the study that the statement the respondents believed was the most accurate about the behavior of children in the age group 3–6 was “children aged between 5 and 6 years may try to touch their mothers’ or other women’s breasts” (96.2 %); the statement they believed was the least accurate was “Sexuality and sex education starts during adolescence” (71.1 %). Aral et al. [30] found that most of the parents in their study believed that sexual education should start in the pre-school period. This finding is consistent with our results. The data collected from the parents of children in the age group 4–6 by Eser and Çeliköz [31] in their study on “The effects of parental attitudes toward the development of sexual identity in the development of a child’s sexual identity” indicated that 78.7 % of the children had not tried to touch the bodies of adults.

In the study, it was determined that the number of correct answers given by the respondents regarding children’s sexual development and sex education was affected by age group, marital status, the status of having children, educational status, and occupation. In parallel to increases in age, increases in education and experience as well as being married and having children are believed to cause an increase in sensitivity toward the importance of child development. The fact that 93 % of our cases thought it was necessary to provide sex education for children supports our assumption. Another finding explaining this result is that considering the necessity of sex education for children and the status of talking to children on any topic related to sexuality affected the number of correct answers given by our respondents pertaining to children’s sexual development and sex education.

In the study, 78.6 % of the cases regarded the statement “In children aged between 5 and 6 years, girls’ questions about sexuality should be answered by mothers and boys’ questions should be answered by fathers” as true. In previous studies, it was demonstrated that mothers talked about sexuality to female children and fathers to male children more frequently [32, 33]. These results support our study findings.

In the study, the statement “If you encounter a scene that includes sexuality while you are watching television with your child, possible solutions for this situation are turning off the television, switching the channel or closing your child’s eyes” was regarded as true by 86.2 % of the respondents and as false by 7.5 %. Attitudes toward topics related to sexuality within the family may affect the development of sexual identity. Excessive supervision can lead to sexual fears and the development of a sexual identity that is characterized by shyness [26].

In the study, the statement “Children do not have sexual instincts” was regarded as true by 23.3 % of the respondents and as false by 61 %. Children start experiencing sexuality at an early age [34]. Freud argued that we possess the two basic instincts of death and sexuality at birth and that the sexual instinct is a part of the instinct to live [25, 35].

In the study, the statement “Parents are more responsible than schools in providing children with sex education” was regarded as true by 84.3 % of the respondents and as false by 8.2 %. On the other hand, the statement “Mothers and fathers should provide children with their first information on sexuality” was regarded as true by 94.3 % and as false by 5.7 %. A great amount of health education begins in the family. In annual surveys, children identify their parents as an important source of sex education [29]. There are findings indicating that talking about sex with parents affects children’s sexual behavior during adolescence. It was reported that young people who received sex education at home started having sex later and engaged less in risky behaviors [36, 37]. In a previous study, it was determined that parents wanted to talk to their children about sexual behavior but they felt embarrassed and uncomfortable and thought that they did not have the skills or the information to talk about sex [38].

## Conclusion and Recommendations

The scope of our study included doctors and nurses, who are in an important position to provide parents with information on children’s sexual development and sex education and are a group that parents regard as sources of reference. Approximately all of our respondents thought that it is necessary to provide children with sex education, and the majority thought that sex education should be provided for children between the ages 7–12. However, in a review of our study findings, we observed that culture had an important impact on sex-related approaches and that embarrassment and shyness is very common. Due to the small number of people who receive training on sex education, approaches to sexuality and sex education are mainly affected by cultural mores. We therefore recommend that health professionals be provided with training programs on “Sex education in children ages 0–6 years.”
